# Increased medial anterior tibial translation and reduced tibiofemoral rotation are observed during weight bearing in anterior cruciate ligament‐deficient knees: A paired within‐subject imaging analysis

**DOI:** 10.1002/jeo2.70876

**Published:** 2026-08-03

**Authors:** Riccardo D'Ambrosi, Stefano Fusco, Filippo Rinciari, Pietro Marchetti, Domenico Albano, Salvatore Gitto, Luca Maria Sconfienza

**Affiliations:** ^1^ IRCCS Ospedale Galeazzi – Sant'Ambrogio Milan Italy; ^2^ Link Campus University Rome Italy; ^3^ Dipartimento di Scienze Biomediche per la Salute Università degli Studi di Milano Milan Italy; ^4^ Sezione di Scienze Radiologiche BIND, Università degli Studi di Palermo Palermo Italy; ^5^ Università degli Studi di Milano, Dipartimento di Scienze Biomediche, Chirurgiche ed Odontoiatriche Milan Italy; ^6^ Dipartimento di Radiologia ASST Grande Ospedale Metropolitano Niguarda Milan Italy

**Keywords:** anterior cruciate ligament, anterior tibial translation, joint instability, knee kinematics, tibiofemoral rotation, weight‐bearing CT

## Abstract

**Purpose:**

To evaluate the effect of weight‐bearing computed tomography (WBCT) on knee kinematics in anterior cruciate ligament (ACL)‐deficient knees using a within‐subject comparison between WBCT and non–weight‐bearing conditions (NWBCT).

**Methods:**

This was a prospective within‐subject observational imaging study. Patients with chronic ACL deficiency underwent both WBCT and NWBCT cone‐beam computed tomography scans of the same knee. Femorotibial rotation (FTR), medial and lateral anterior tibial translation (ATT), and trochlear groove–patellar tendon angle (FTR 2‐PTTG) were measured using standardised multiplanar reconstructions. Interobserver reliability was assessed using intraclass correlation coefficients (ICC), while paired *t*‐tests were used to compare measurements between WBCT and NWBCT conditions.

**Results:**

A total of 16 patients (mean age 35.6 ± 10.2 years) were included. WBCT conditions resulted in a significant reduction in FTR compared to NWBCT (3.83 ± 3.30 vs. 8.50 ± 5.10 degrees; *p* = 0.011) and a significant increase in medial ATT (5.52 ± 3.30 vs. 2.83 ± 3.68 mm; *p* = 0.021). No significant differences were observed for lateral ATT or FTR 2‐PTTG. Interobserver reliability was good to excellent for most measurements (ICC range 0.87–0.98), whereas FTR 2‐PTTG demonstrated moderate interobserver reliability (ICC range 0.59–0.71). Correlation analysis revealed a strong positive association between FTR and lateral ATT under NWBCT conditions (*r* = 0.85, *p* < 0.001).

**Conclusions:**

WBCT significantly alters knee kinematics in ACL‐deficient knees, resulting in increased medial ATT and reduced FTR compared with NWBCT conditions. These findings suggest that conventional imaging may not fully reflect knee kinematics under physiological loading conditions.

**Level of Evidence:**

Level II, prospective within‐subject observational imaging study.

**Abbreviations**
ACL: anterior cruciate ligament
ALL: anterolateral ligament
ATT: anterior tibial translation
CBCT: cone‐beam computed tomography
CI: confidence intervals
CT: computed tomography
FTR: femorotibial rotation angle
FTR 2‐PTTG: trochlear groove–patellar tendon angle
ICC: intraclass correlation coefficient
MRI: magnetic resonance imaging
NWBCT: non–weight‐bearing computed tomography
SD: standard deviation
STROBE: Strengthening the Reporting of Observational Studies in Epidemiology
WB: weight‐bearing
WBCT: weight‐bearing computed tomography

## INTRODUCTION

Anterior cruciate ligament (ACL) deficiency is associated with altered knee biomechanics, including increased anterior tibial translation (ATT) and abnormal tibiofemoral rotation, which contribute to joint instability and may predispose to early degenerative changes [13, 30]. Objective quantification of these alterations is essential for understanding knee kinematics and guiding clinical decision‐making [16]. Although clinical tests such as the Lachman and pivot‐shift manoeuvres remain the cornerstone of ACL assessment, their interpretation is partly dependent on examiner experience [4, 5]. Consequently, imaging techniques have been increasingly adopted to provide a reproducible and quantitative evaluation of knee instability through the assessment of ATT and rotational parameters [22, 26].

Magnetic resonance imaging (MRI) and conventional computed tomography (CT) are widely used in clinical practice; however, both are typically performed in supine, non–weight‐bearing conditions (NWBCT). This represents a significant limitation, as these modalities do not reproduce physiological loading, which plays a crucial role in determining joint alignment, joint laxity, contact mechanics, and rotational behaviour. Consequently, static imaging may underestimate the true extent of instability in ACL‐deficient knees [7, 23].

In recent years, weight‐bearing computed tomography (WBCT) has emerged as a promising imaging modality capable of evaluating the lower limb under physiological load, providing high‐resolution three‐dimensional assessment of joint alignment and kinematics. Previous studies have demonstrated the feasibility of WBCT in assessing ACL‐deficient knees and have shown increased ATT and tibiofemoral rotation compared to the contralateral healthy knee [19, 33].

However, current evidence is primarily based on comparisons between injured and uninjured knees, which may be influenced by inter‐individual variability and do not isolate the specific effect of loading conditions [19, 33].

Therefore, the aim of the present study was to evaluate the effect of WBCT on knee kinematics in ACL deficient knees using a within‐subject comparison between WBCT and NWBCT. It was hypothesised that physiological loading would significantly influence tibiofemoral kinematics, resulting in measurable differences in ATT and rotational parameters compared to unloaded conditions.

## MATERIALS AND METHODS

### Study design and patient selection

This was a prospective within‐subject observational imaging study. This study was conducted in accordance with the Strengthening the Reporting of Observational Studies in Epidemiology (STROBE) declaration [29]. All procedures were conducted in compliance with the principles outlined in the 1964 Declaration of Helsinki and its subsequent amendments [9].

The present study was approved by the Ethics Committee of the IRCCS, Ospedale San Raffaele (ACL‐L2104).

This was a prospective, diagnostic study conducted between January 2025 and January 2026. Patients were eligible for inclusion if they were between 18 and 54 years of age, had a complete ACL tear documented by MRI, and presented with a chronic injury defined as a time from trauma of at least 6 months. Additional inclusion criteria included the absence of prior surgical procedures on the examined knee, the ability to undergo WBCT in a standing position, and the provision of written informed consent. Only skeletally mature patients were included. Patients were excluded in the presence of bilateral ACL injuries, joint infections or septic arthritis, or advanced osteoarthritis defined as Kellgren–Lawrence grade ≥3. Additional exclusion criteria included the inability to maintain a standing position during WBCT, lack of informed consent, and skeletal immaturity.

### Imaging acquisition

All examinations were performed using a dedicated extremity cone‐beam computed tomography (CBCT) system (Carestream OnSight 3D), with a slice thickness of 0.45 mm.

Each patient underwent two scans: one in a standing WBCT position and one under NWBCT conditions, with the patient seated on a dedicated chair and the knee positioned within a gantry inclined at 90°. In both WBCT and NWBCT acquisitions, the knee was maintained in full extension. Therefore, the only difference between the two imaging conditions was the presence or absence of physiological weight‐bearing (WB) load. All images were analysed using the institutional PACS workstation (Sectra IDS7), which allowed standardised measurements to be performed on multiplanar reconstructions for all subjects.

### CT measurement protocol

The images were independently evaluated by two readers who were not blinded to the WBCT and NWBCT acquisition conditions: a radiology resident with 1 year of experience in musculoskeletal imaging and a fifth‐year orthopaedic resident.

Prior to the study analysis, both readers underwent a dedicated training session supervised by an experienced musculoskeletal radiologist, using a separate dataset of knee WBCT examinations.

After this training, the two readers independently measured lateral and medial ATT, as well as two angular measurements of femorotibial rotation (FTR), the FTR and the trochlear groove–patellar tendon angle (FTR 2‐PTTG), and medial and lateral tibial slope.

All measurements were performed under both WBCT and NWBCT conditions, except for medial and lateral tibial slope, which were assessed only on WBCT.

### Medial and lateral ATT

**Figure 1:**
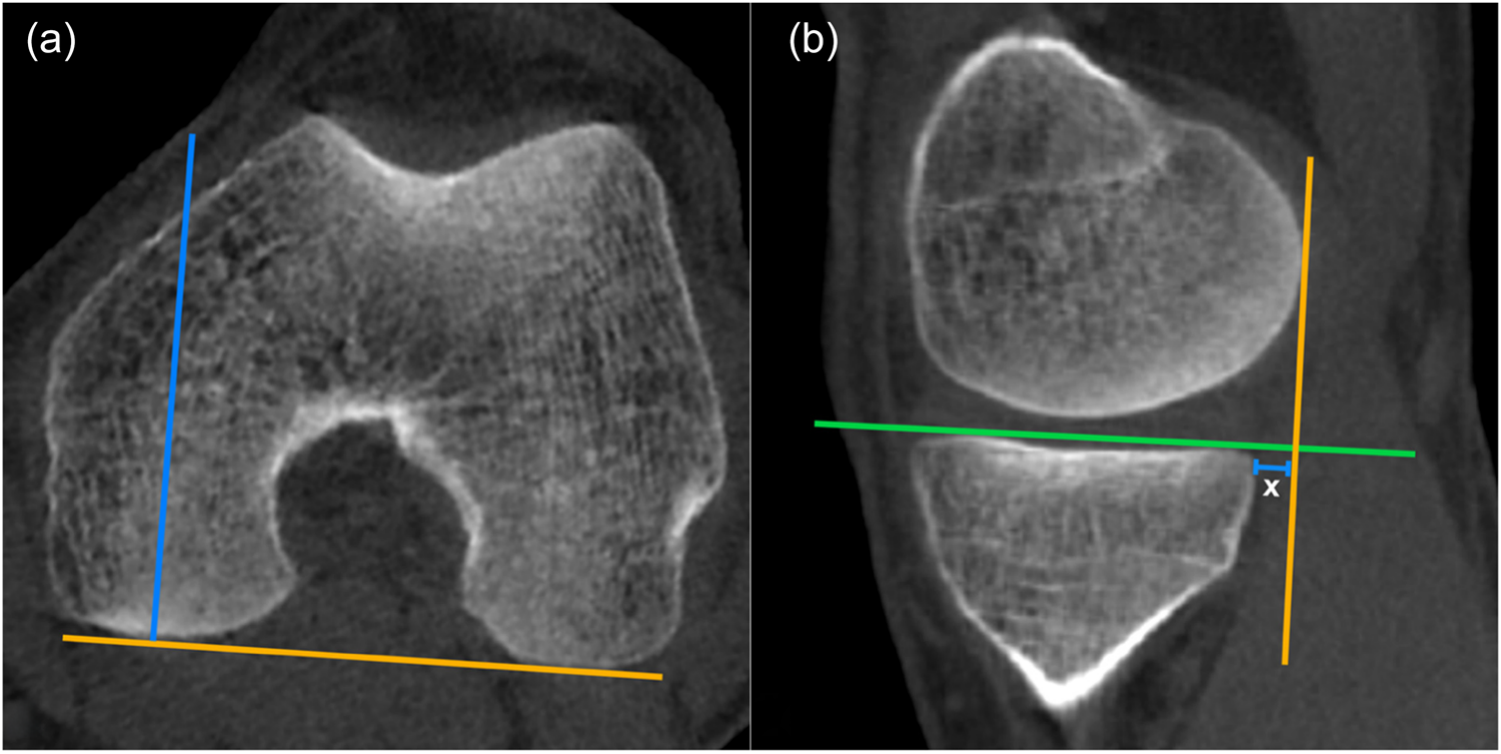
Measurement of anterior tibial translation on sagittal‐reformatted images. (a) On the axial image, the midportion of the femoral condyle is identified (blue line). (b) Corresponding sagittal image. The anterior tibial translation (x) is the distance between the posterior margin of the tibial plateau and the perpendicular line tangent to the posterior profile of the femoral condyle (yellow line). The green line represents the tibial plateau plane.

The measurement was defined as the distance between a line drawn perpendicular to the posterior margin of the tibial plateau and the most posterior point of the femoral condyles on sagittal images obtained at the centre of the medial and lateral femoral condyles (Figure 1) [19, 33].

### FTR angle

**Figure 2:**
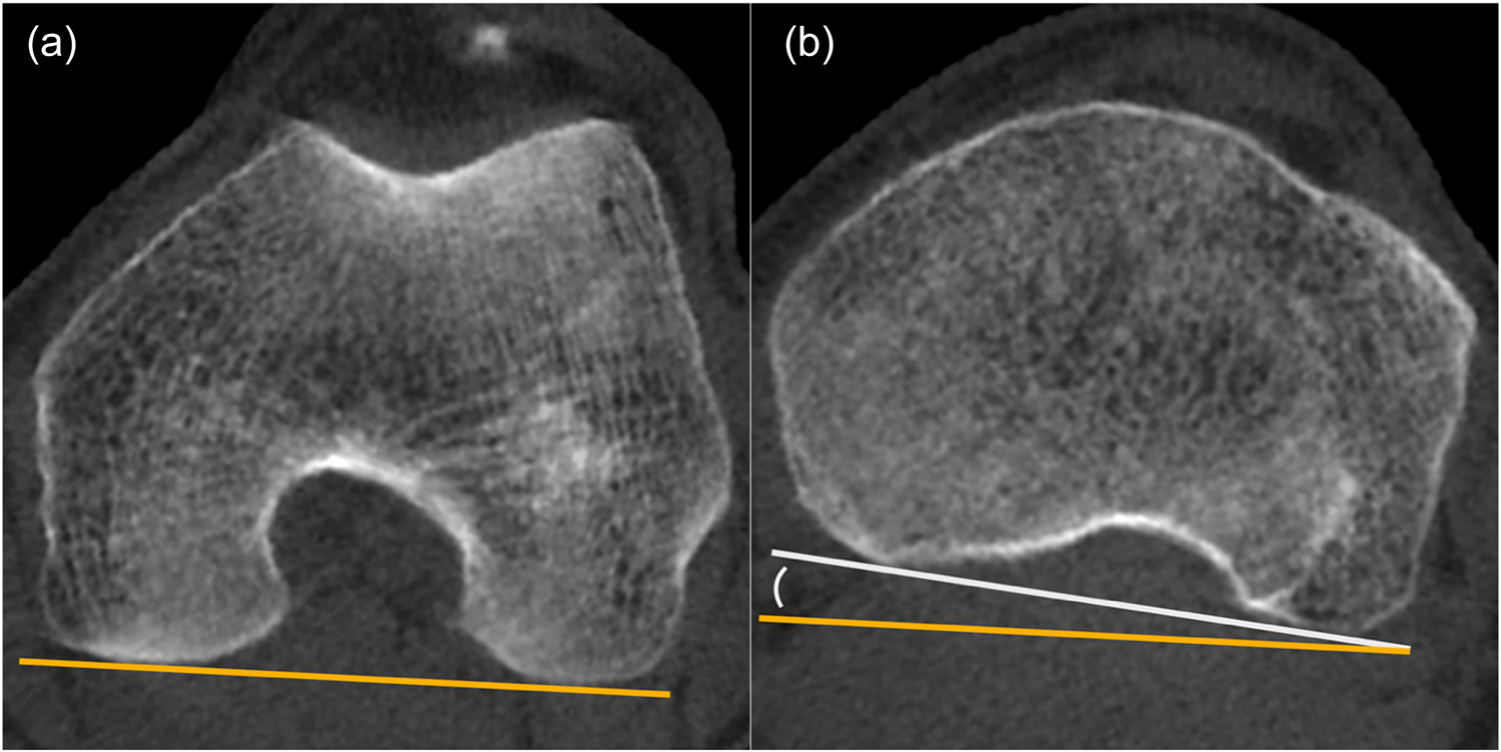
Measurement of femorotibial rotation angle (FTR) on axial images. (a) Axial image at the transepicondylar level. The yellow line is tangent to the posterior aspect of the femoral condyles. (b) Axial image, one slice above the proximal margin of the fibular head. The white line is tangent to the posterior tibial plateau. FTR is defined as the angle between the yellow and the white lines.

The angle was defined as the angle between a line tangent to the posterior contour of the tibial plateau, measured one slice cranial to the fibular head, and a line tangent to the posterior femoral condyles on axial images obtained at the level of the transepicondylar line (Figure 2) [19, 33].

### FTR/patellar tendon‐trochlear groove angle (FTR 2‐PTTG)

To assess FTR 2‐PTTG, a tangent line to the posterior profile of the femoral condyles was drawn at the level of the transepicondylar line. A second line, perpendicular to the posterior femoral condylar line and passing through the trochlear groove (trochlear line), was then traced.

**Figure 3:**
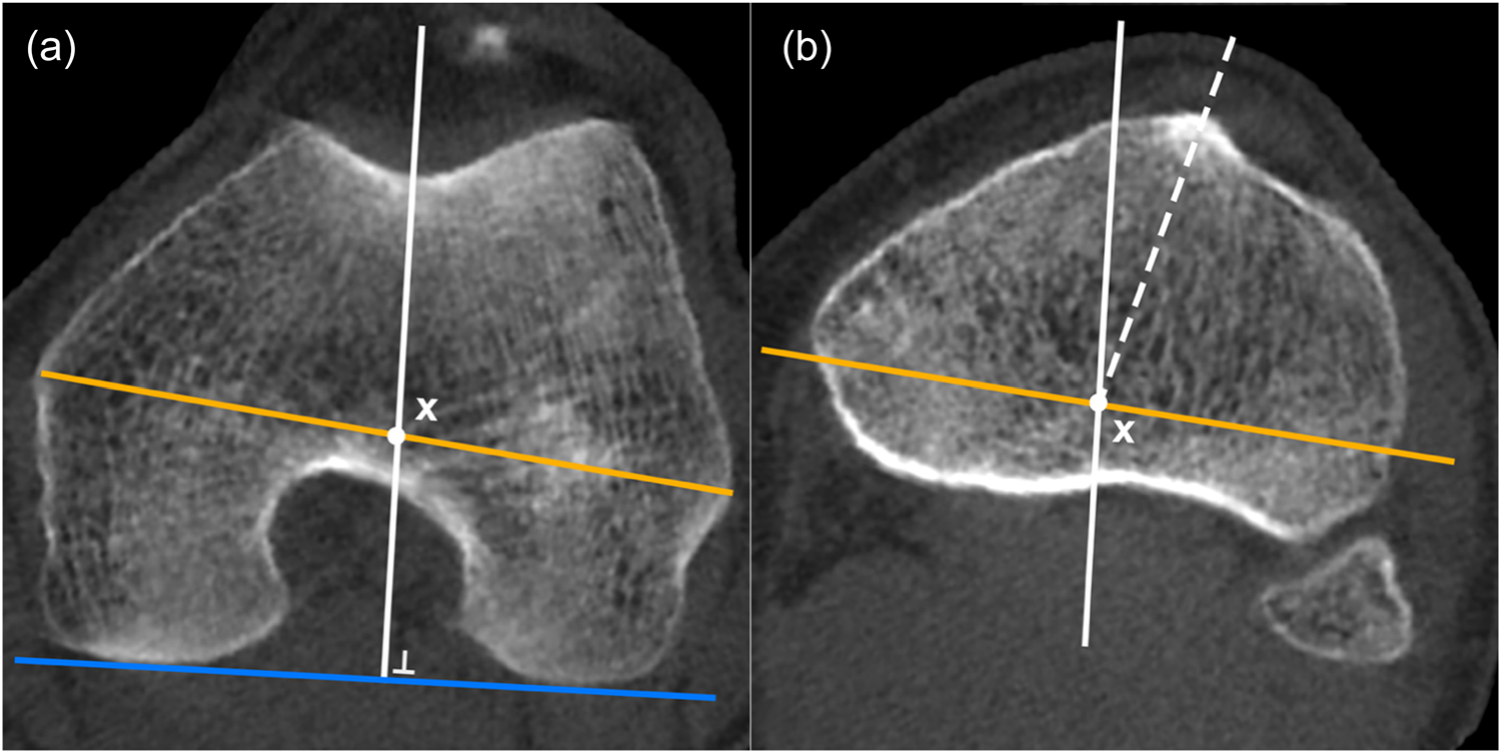
Measurement of femorotibial rotation/patellar tendon‐trochlear groove (FTR 2‐PTTG) angle on axial images. (a) Axial image at the transepicondylar level. Yellow line: transepicondylar line; blue line: posterior femoral condylar line; white line: trochlear line (perpendicular to the posterior femoral condylar line and passing through the trochlear groove). The intersection between transepicondylar and trochlear lines defines the centre of the knee (x). (b) Axial image at the level of the tibial tuberosity. The white dotted line passes through the midpoint of the patellar tendon insertion and the knee centre. FTR 2‐PTTG is defined as the angle between the trochlear line (white) and the patellar tendon line (dotted white).

The intersection between the trochlear line and the transepicondylar line was defined as the knee centre. These reference lines were subsequently transferred to the level of the tibial tuberosity, at the midpoint of the patellar tendon insertion. The angle between the trochlear line and a line connecting the knee centre to the centre of the patellar tendon insertion was defined as FTR 2‐PTTG (Figure 3) [19, 33].

### Medial and lateral tibial slope

On a central sagittal image, two circles were positioned within the tibia. The proximal circle was placed to be tangent to the anterior, posterior, and superior cortices, whereas the distal circle was tangent to the anterior and posterior cortices. The centre of the distal circle was positioned on the circumference of the proximal circle to standardise the distance between the two circles. The tibial longitudinal axis was defined as the line connecting the centres of the two circles. At the level of the medial and lateral tibial plateaus, the tibial slope was defined as the angle between a line tangent to the tibial plateau and a line perpendicular to the tibial longitudinal axis.

**Figure 4:**
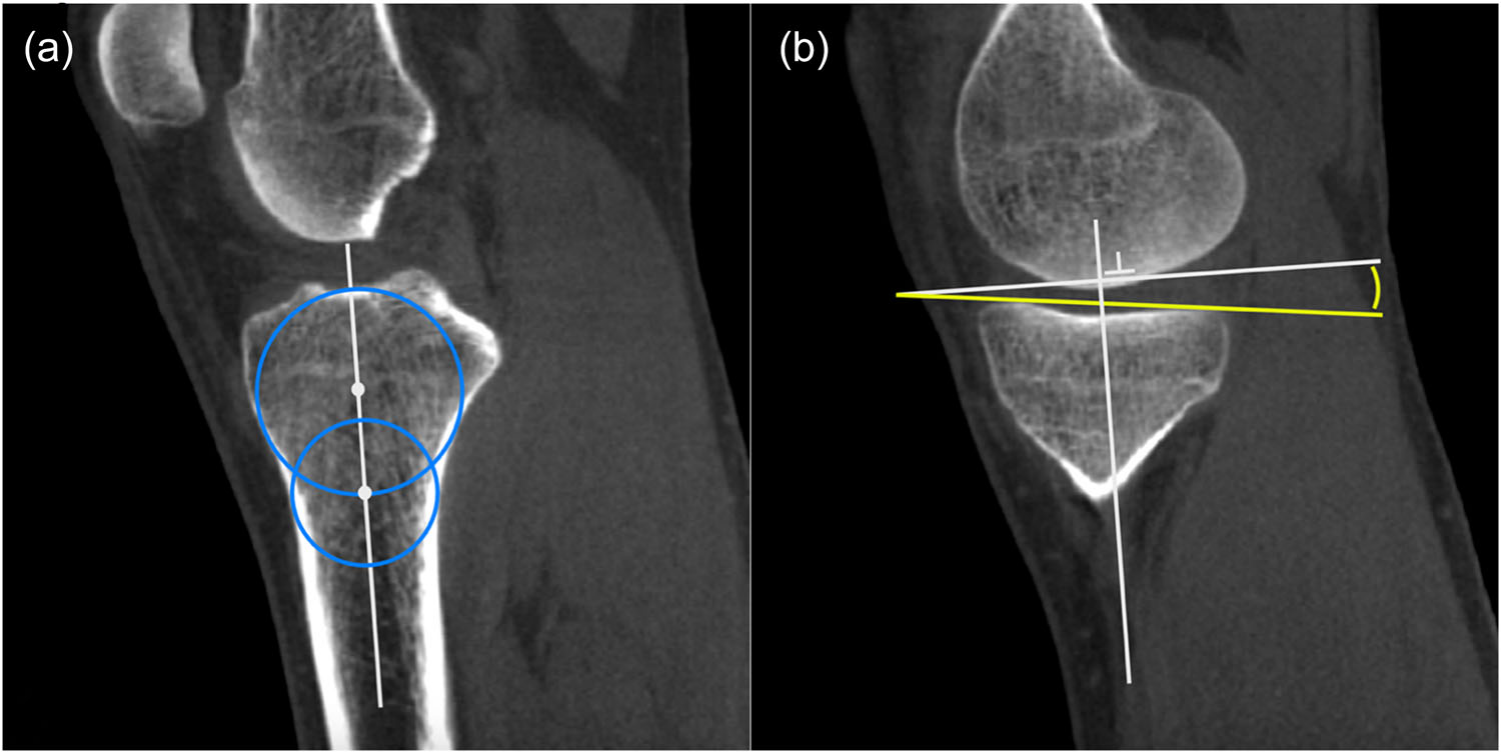
Measurement of tibial slope using the two‐circle method on sagittal‐reformatted images. (a) On the central sagittal image, two reference circles are drawn within the proximal tibia. The proximal circle is adjusted to contact the anterior, posterior, and superior cortices, whereas the distal circle is adjusted to contact the anterior and posterior cortices. The centre of the distal circle is positioned on the circumference of the proximal circle, ensuring a standardised distance between the two circles. The tibial longitudinal axis is then defined as the line joining their centres. (b) On the sagittal image crossing the midpoint of the medial tibial plateau, tibial slope is calculated as the angle formed between a tangent to the tibial plateau and a line perpendicular to the tibial longitudinal axis.

These measurements were performed only on the WBCT images, as tibial slope is an intrinsic anatomical characteristic of the proximal tibia and is not expected to differ between WBCT and NWBCT conditions (Figure 4) [12, 15, 18].

### Reliability assessment and statistical analysis

Summary statistics were presented as means and standard deviation (SD) or absolute frequencies and percentages. Interrater reliability was assessed with the intraclass correlation coefficients (ICCs) calculated with the respective 95% confidence intervals (CIs). The ICC was computed by a multiple‐rating, absolute‐agreement, two‐way random‐effects model with two raters across 16 subjects. ICC values were interpreted as follows: less than 0.50 indicated poor reliability; between 0.50 and 0.75 indicated moderate reliability; between 0.75 and 0.90 indicated good reliability; and greater than 0.90 indicated excellent reliability [17].

To compare measures between NWBCT *and WBCT*, we first compared the average value between the two observers. Second, values NWBCT *and WBCT* were compared and tested with paired *t*‐tests. Subgroup analyses comparing NWBCT *vs. WBCT* among patients with the same sex and similar age (above and below the rounded average value) were also performed with paired *t*‐tests (Appendix S1).

Pearson correlation coefficients were also estimated and tested to explore correlation among continuous variables. Pearson correlation coefficients were calculated from the mean values of the two observers. All tests were two‐sided and *p*‐value less than 0.05 was taken as statistically significant. Statistical analyses were conducted in R version 4.1.1.

### Sample size

A sample size calculation was performed to determine the number of subjects required for the interrater reliability using the ICC. It was assumed an expected ICC of 0.95 and a minimum acceptable ICC of 0.80, with two observers, a two‐sided significance level of 0.05 and a statistical power of 80%. Under these assumptions, a minimum of 16 subject was required [33].

## RESULTS

**Figure 5:**
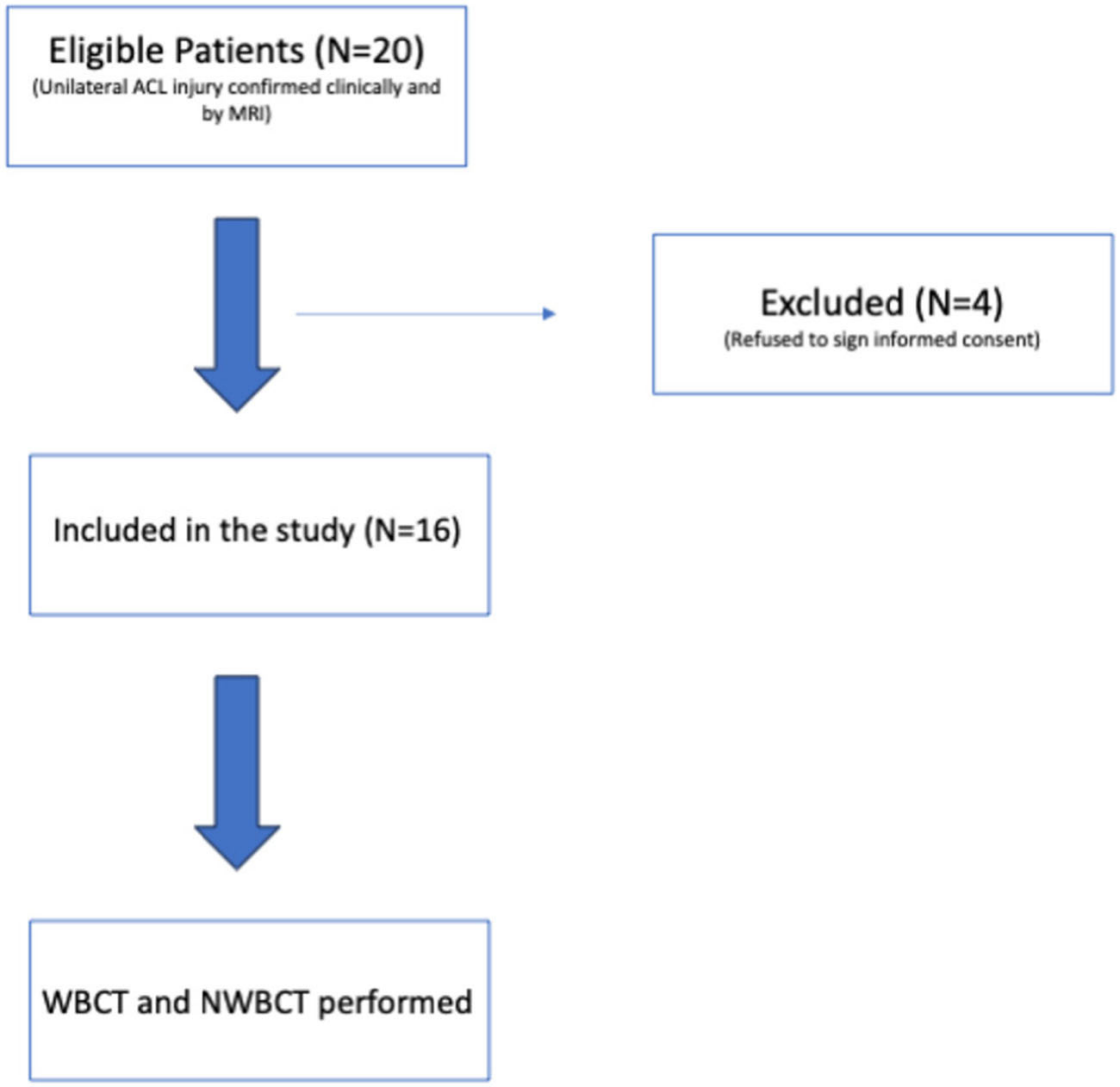
Flow diagram of patient inclusion. Sixteen patients with ACL deficiency underwent both WBCT and NWBCT for the assessment of ATT and FTR and FTR 2‐trochlear groove–patellar tendon angle (PTTG). ACL, anterior cruciate ligament; ATT, anterior tibial translation; FTR, femorotibial rotation angle; MRI, magnetic resonance imaging; NWBCT, non‐weight‐bearing computed tomography; WBCT, weight‐bearing computed tomography.

A total of 20 patients were initially recruited. Of these, four were excluded due to lack of informed consent, resulting in 16 patients included in the study. The participant selection process is illustrated in the flowchart in Figure 5.

The mean age of the included subjects was 35.56 ± 10.19 years. The cohort consisted of 10 male (62.5%) and 6 females (37.5%). Regarding laterality, ACL injury involved the left knee in nine patients (56.2%) and the right knee in seven patients (43.8%).

### Interobserver reliability

Interobserver reliability was overall good to excellent across most measurements. Under NWBCT conditions, excellent agreement was observed for FTR (ICC 0.94; 95% CI 0.83–0.98; *p* < 0.001) and medial ATT (ICC 0.93; 95% CI 0.80–0.98; *p* < 0.001), while lateral ATT showed good reliability (ICC 0.89; 95% CI 0.69–0.96; *p* < 0.001). In contrast, FTR 2‐PTTG demonstrated moderate agreement (ICC 0.59; 95% CI −0.22 to 0.86; *p* = 0.053).

Under WBCT conditions, reliability remained good to excellent for all primary measurements, with ICC values of 0.87 for FTR, 0.95 for medial ATT, and 0.98 for lateral ATT (all *p* < 0.001). FTR 2‐PTTG again showed lower agreement (ICC 0.71; 95% CI −0.05 to 0.91; *p* = 0.051) (Table 1).

**Table 1:** Interobserver agreement for FTR1, ATT and FTR‐2 PTTG measurements in weight‐bearing and non–weight‐bearing conditions.

	Observer 1 *N* = 16 Mean ± SD	Observer 2 *N* = 16 Mean ± SD	*t*‐test *p*‐value	ICC (95% CI)	ICC *p*‐value
NWBCT					
FTR (degrees)	8.19 ± 5.21	8.81 ± 5.29	0.744	0.94 (0.83; 0.98)	<0.001*
FTR 2‐PTTG (degrees)	20.85 ± 6.76	21.13 ± 8.83	0.920	0.59 (−0.22; 0.86)	0.053
Medial ATT (mm)	3.06 ± 3.66	2.59 ± 3.96	0.727	0.93 (0.8; 0.98)	<0.001*
Lateral ATT (mm)	2.54 ± 5.83	3.06 ± 6.26	0.812	0.89 (0.69; 0.96)	<0.001*
WBCT					
FTR (degrees)	3.16 ± 2.76	4.51 ± 4.03	0.278	0.87 (0.55; 0.96)	<0.001*
FTR 2‐PTTG (degrees)	25.74 ± 4.58	21.69 ± 6.00	0.040*	0.71 (−0.05; 0.91)	0.051
Medial ATT (mm)	5.84 ± 3.77	5.20 ± 2.93	0.597	0.95 (0.84; 0.98)	<0.001*
Lateral ATT (mm)	1.79 ± 4.92	2.74 ± 4.90	0.589	0.98 (0.87; 0.99)	<0.001*
Tibial slope					
Medial tibial slope angle (degrees)	6.32 ± 4.37	6.51 ± 3.33	0.889	0.95 (0.86; 0.98)	<0.001*
Lateral tibial slope angle (degrees)	9.17 ± 5.79	7.86 ± 3.22	0.437	0.8 (0.46; 0.93)	0.001*

* Statistical significant difference.

Abbreviations: ATT, anterior tibial translation; FTR 2‐PTTG, trochlear groove–patellar tendon angle FTR, femorotibial rotation angle; ICC, intraclass correlation coefficient; NWBCT, non–weight‐bearing computed tomography; WBCT, weight‐bearing computed tomography.

Interobserver reliability of all measured parameters, including FTR, ATT and trochlear groove–patellar tendon angle (FTR 2‐PTTG), assessed under both weight‐bearing and NWBCT. Agreement between observers is reported as ICCs with 95% CIs. *p*‐Values refer to statistical significance of agreement.

### Comparison between NWBCT and WBCT conditions

Significant differences were observed between NWBCT and WBCT conditions for selected parameters. FTR was significantly lower under WBCT conditions compared to NWBCT (3.83 ± 3.30 vs. 8.50 ± 5.10 degrees; *p* = 0.011). Conversely, medial ATT was significantly higher under WBCT (5.52 ± 3.30 vs. 2.83 ± 3.68 mm; *p* = 0.021).

No significant differences were observed for FTR 2‐PTTG (23.71 ± 4.91 vs. 20.99 ± 6.60 degrees; *p* = 0.113) or lateral ATT (2.27 ± 4.88 vs. 2.80 ± 5.73 mm; *p* = 0.780) (Table 2).

**Table 2:** Within‐subject comparison of FTR and anterior tibial translation under non–weight‐bearing and weight‐bearing conditions.

	NWBCT *N* = 16 Mean ± SD	WBCT *N* = 16 Mean ± SD	*p*‐value
FTR (degrees)	8.50 ± 5.10	3.83 ± 3.30	0.011*
FTR 2‐PTTG (degrees)	20.99 ± 6.60	23.71 ± 4.91	0.113
Medial ATT (mm)	2.83 ± 3.68	5.52 ± 3.30	0.021*
Lateral ATT (mm)	2.80 ± 5.73	2.27 ± 4.88	0.780

* Statistical significant difference.

Abbreviations: ATT, anterior tibial translation; FTR 2‐PTTG, trochlear groove–patellar tendon angle FTR, femorotibial rotation angle; NWBCT, non–weight‐bearing computed tomography; WBCT, weight‐bearing computed tomography.

Comparison of FTR, trochlear groove–patellar tendon angle (FTR 2‐PTTG), and ATT between NWBCT and WBCT in ACL‐deficient knees. Values are expressed as mean ± SD. *p*‐Values refer to paired comparisons between conditions.

### Correlations

Significant correlations were observed among several measured parameters. Under NWBCT conditions, a strong positive correlation was found between FTR and lateral ATT (*r* = 0.85; *p* < 0.001). Medial ATT was moderately correlated with the lateral tibial slope angle (*r* = 0.60; *p* = 0.014). In addition, FTR showed a moderate negative correlation with FTR 2‐PTTG (*r* = −0.62; *p* = 0.011).

Under WBCT conditions, medial ATT demonstrated a moderate positive correlation with the lateral tibial slope angle (*r* = 0.53; *p* = 0.035), and FTR 2‐PTTG showed a moderate negative correlation with lateral ATT (*r* = −0.53; *p* = 0.036).

Age was moderately correlated with FTR 2‐PTTG under WBCT conditions (*r* = 0.55; *p* = 0.027). Additionally, a moderate positive correlation was observed between medial and lateral tibial slope angles (*r* = 0.53; *p* = 0.034) (Table 3).

**Table 3:** Pearson correlation analysis of kinematic parameters under weight‐bearing and non–weight‐bearing conditions.

Variables		Correlation coefficient	*p*‐value
FTR NWBCT	Lateral ATT NWBCT	0.85	<0.001*
Medial ATT NWBCT	Lateral tibial slope	0.60	0.014*
Age	FTR 2‐PTTG WBCT	0.55	0.027*
Medial tibial slope	Lateral tibial slope	0.53	0.034*
Medial ATT WBCT	Lateral tibial slope	0.53	0.035*
FTR 2‐PTTG NWBCT	Lateral ATT NWBCT	−0.51	0.045*
FTR 2‐PTTG WBCT	Lateral ATT WBCT	−0.53	0.036*
FTR NWBCT	FTR 2‐PTTG NWBCT	−0.62	0.011*
Medial ATT NWBCT	Lateral ATT WBCT	−0.69	0.003*

* Statistical significant difference.

Abbreviations: ATT, anterior tibial translation; FTR 2‐PTTG, trochlear groove–patellar tendon angle FTR, femorotibial rotation angle; NWBCT, non–weight‐bearing computed tomography; WBCT, weight‐bearing computed tomography.

Significant correlations between measured variables, including FTR, ATT, FTR 2‐PTTG, and tibial slope angles. Correlation coefficients (*r*) are reported based on Pearson correlation analysis. Positive values indicate direct correlations, while negative values indicate inverse relationships. *p*‐Values refer to statistical significance.

## DISCUSSION

The most important finding of the present study is that WBCT conditions significantly alter knee kinematics in ACL‐deficient knees compared with non‐weight bearing condition, resulting in a reduction in FTR and an increase in medial ATT. These findings support the concept that conventional NWBCT imaging may not fully reflect knee biomechanics under physiological loading conditions.

Although FTR 2‐PTTG showed moderate interobserver reliability (ICC 0.59–0.71), lower than that observed for the other assessed parameters, it was retained because it provides complementary information regarding the relationship between rotational alignment and patellofemoral morphology. Nevertheless, its reproducibility remains suboptimal, and further refinement and validation of this measurement are warranted before widespread clinical adoption.

The importance of evaluating knee mechanics under physiological load has been increasingly emphasised in recent years. Conventional imaging modalities, including MRI and CT, are typically performed in NWBCT conditions and may therefore fail to reproduce in vivo joint behaviour. WB MRI studies have demonstrated that imaging under load can reveal biomechanical alterations that are not detectable in standard supine conditions [2, 14, 20, 28]. Similarly, CT‐based investigations have shown that transitioning from NWBCT to WBCT conditions leads to measurable changes in joint alignment, rotation, and displacement parameters [3, 6, 27].

WBCT has emerged as a valuable modality for evaluating knee kinematics in three dimensions under physiological conditions [6, 24, 27, 28, 31]. WBCT enables assessment of joint space, alignment, and contact mechanics, which are significantly influenced by loading conditions [21]. However, the lack of standardised acquisition protocols and measurement methods remains a limitation, potentially affecting reproducibility and comparability across studies [1].

Previous WBCT studies have primarily focused on inter‐limb comparisons. Leão et al. demonstrated increased ATT and FTR in ACL‐deficient knees compared to the contralateral side across multiple loading conditions [19]. Similarly, Zelada et al. reported significantly increased ATT and altered rotational parameters in ACL‐injured knees under WBCT conditions, confirming the ability of WBCT to detect instability patterns [33].

However, these inter‐limb comparisons do not isolate the effect of loading itself. The present study addresses this limitation by adopting a within‐subject design, directly comparing WBCT and NWBCT conditions in the same ACL‐deficient knee. This approach allows a more precise evaluation of the biomechanical impact of axial loading, eliminating inter‐individual variability.

A key finding of the present study is the selective increase in medial ATT under WB conditions. Previous studies have reported increased ATT in ACL‐deficient knees under loading [33], but without distinguishing compartment‐specific behaviour. The present findings suggest that loading may accentuate a predominantly medial instability pattern. This is consistent with evidence showing that WB conditions influence joint contact distribution and may reveal biomechanical alterations not detectable in unloaded imaging [8].

Conversely, tibiofemoral rotation was found to decrease under WB conditions. This finding contrasts with previous WBCT studies reporting increased rotational values in ACL‐deficient knees [19], but this discrepancy is likely explained by differences in study design. Inter‐limb comparisons reflect the overall effect of ACL deficiency, whereas within‐subject comparisons isolate the effect of loading alone.

From a biomechanical perspective, this observation may be explained by the role of secondary stabilisers, particularly the anterolateral structures of the knee. The anterolateral ligament (ALL) has been shown to function as a passive restraint to internal rotation, especially in ACL‐deficient knees [32]. Under WBCT conditions, increased joint compression and activation of these secondary stabilisers may limit rotational motion while allowing anterior translation, potentially explaining the reduction in FTR observed in the present study.

Furthermore, WBCT conditions may activate physiological mechanisms such as the screw‐home movement, characterised by external rotation of the tibia relative to the femur during extension. This phenomenon has been demonstrated in upright CT studies and may influence rotational measurements obtained under load [25]. These findings further support the concept that rotational kinematics are highly dependent on loading conditions and joint compression.

Interestingly, no significant differences were observed for lateral ATT or for the FTR 2‐PTTG, suggesting that not all kinematic parameters are equally sensitive to loading conditions. This variability is consistent with previous studies highlighting the complexity of rotational measurements and the need for standardised protocols in WBCT analysis [1, 6, 25, 31].

The correlation analysis further supports the biomechanical consistency of the findings. The association between rotational and translational parameters reflects the complex interplay between different components of knee instability. Additionally, previous studies have shown that joint alignment and kinematics are strongly influenced by WB conditions and flexion angle, further emphasising the importance of evaluating the knee under physiological loading [10, 11].

From a clinical perspective, these findings suggest that non–WB imaging may underestimate functional instability in ACL‐deficient knees. The integration of WB imaging techniques into clinical practice may therefore provide a more comprehensive assessment of knee biomechanics and improve the characterisation of instability patterns under physiological loading conditions. Future studies should investigate whether these imaging findings are associated with specific injury patterns or may influence surgical decision‐making, including meniscal preservation strategies and anterolateral augmentation procedures.

### Limitations

This study has several limitations. First, the sample size was relatively small, which may limit the generalisability of the findings and reduce the statistical power to detect small‐to‐moderate effects. Although a priori sample size calculation supported the reliability analysis, the study was not powered for subgroup or exploratory correlation analyses. Consequently, any age‐ or sex‐related differences, as well as secondary associations between measured parameters, should be interpreted with caution and considered hypothesis‐generating. Second, only static imaging conditions were evaluated, and dynamic assessment of knee kinematics was not performed. Therefore, potential changes in tibiofemoral motion occurring throughout the range of motion could not be assessed. Future studies using dynamic or sequential WB imaging techniques may provide additional insights into knee kinematics under physiological loading conditions. Third, although interobserver reliability was generally high, some parameters, particularly FTR 2‐PTTG, demonstrated lower reproducibility. Fourth, the observers were not blinded to the WBCT and NWBCT acquisition conditions, which may have introduced measurement bias. Finally, although the paired within‐subject design allowed each patient to serve as his or her own control, comparison with healthy knees was not performed, precluding direct comparison with normal knee biomechanics. Furthermore, the correlation analyses were exploratory in nature and were not adjusted for multiple comparisons; therefore, these findings should be interpreted as hypothesis‐generating rather than confirmatory. Future studies including appropriately selected control populations and larger sample sizes are warranted to validate and further contextualise the observed kinematic alterations.

## CONCLUSION

WBCT significantly alters knee kinematics in ACL‐deficient knees, resulting in increased medial ATT and reduced FTR compared with NWBCT conditions. These findings suggest that conventional imaging may not fully reflect knee kinematics under physiological loading conditions.

## AUTHOR CONTRIBUTIONS

**Riccardo D'Ambrosi**: Conception; design; writer; materials; data collection; literature review. **Stefano Fusco**: Design; data collection; processing; analysis; writer; materials; literature review. **Filippo Rinciari**: Conception; design; writer; materials; data collection; literature review. **Pietro Marchetti**: Writer; critical review; literature review; processing; interpretation; supervision. **Domenico Albano**: Conception; supervision; interpretation; critical review. **Salvatore Gitto**: Literature review; materials; design; conception; analysis. **Luca Maria Sconfienza**: Conception; supervision; interpretation; critical review.

## CONFLICT OF INTEREST STATEMENT

The authors declare no conflicts of interest.

## ETHICS STATEMENT

The present study was approved by the Ethics Committee of the IRCCS, Ospedale San Raffaele (ACL‐L2104). Informed consent was obtained from all individual participants included in the study.

## Supporting information

Supporting File

## Data Availability

Raw data are available upon request to the corresponding author.
