# Supplementary material for: Increased medial anterior tibial translation and reduced tibiofemoral rotation are observed during weight bearing in anterior cruciate ligament‐deficient knees: A paired within‐subject imaging analysis
Source: J Exp Orthop. 2026 Aug 3;13(3):e70876. doi: 10.1002/jeo2.70876 (PMC13431683; doi:10.1002/jeo2.70876)
Supplement: Supplementary file 1 — Supporting File [file JEO2-13-e70876-s001.docx]

*Appendix 1*

*Subgroup analysis*

When stratified by sex, significant differences between NWBCT and WBCT conditions were observed only in male patients. In this subgroup, FTR was significantly reduced under WBCT conditions (3.10 ± 3.41 vs 7.73 ± 4.06 degrees; p=0.028), while medial ATT was significantly increased (5.24 ± 2.71 vs 1.43 ± 3.88 mm; p=0.013). No significant differences were found for FTR 2–PTTG or lateral ATT (Table S1).

**Table S1.** Comparison of kinematic parameters between non–weight-bearing and weight-bearing conditions in male patients

|  | NWBCT  N=10  Mean ±SD | WBCT  N=10  Mean ±SD | p-value |
| --- | --- | --- | --- |
| FTR (degrees) | 7.73 ±4.06 | 3.10 ±3.41 | 0.028* |
| FTR 2 - PTTG (degrees) | 19.27 ±5.23 | 22.60 ±5.04 | 0.144 |
| Medial ATT (mm) | 1.43 ±3.88 | 5.24 ±2.71 | 0.013* |
| Lateral ATT (mm) | 1.95 ±5.55 | 3.30 ±5.20 | 0.619 |

Subgroup analysis of male patients comparing femorotibial rotation (FTR), trochlear groove–patellar tendon angle (FTR 2-PTTG), and anterior tibial translation (ATT) between non–weight-bearing and weight-bearing conditions. Values are expressed as mean ± standard deviation. P-values refer to paired comparisons between conditions.

In contrast, no statistically significant differences were observed in female patients for any of the evaluated parameters (Table S2).

**Table S2.** Comparison of kinematic parameters between non–weight-bearing and weight-bearing conditions in female patients

|  | NWBCT  N=6  Mean ±SD | WBCT  N=6  Mean ±SD | p-value |
| --- | --- | --- | --- |
| FTR (degrees) | 9.78 ±6.73 | 5.06 ±2.98 | 0.222 |
| FTR 2 - PTTG (degrees) | 23.86 ±8.09 | 25.56 ±4.48 | 0.565 |
| Medial ATT (mm) | 5.16 ±1.78 | 5.98 ±4.36 | 0.654 |
| Lateral ATT (mm) | 4.22 ±6.26 | 0.56 ±4.15 | 0.143 |

Subgroup analysis of female patients comparing femorotibial rotation (FTR), trochlear groove–patellar tendon angle (FTR 2-PTTG), and anterior tibial translation (ATT) between non–weight-bearing and weight-bearing conditions. Values are expressed as mean ± standard deviation. P-values refer to paired comparisons between conditions.

When stratified by age, patients aged ≥35 years showed a significant reduction in FTR under WBCT conditions compared to NWBCT (3.85 ± 3.20 vs 9.07 ± 4.52 degrees; p=0.043). No significant differences were observed for the other parameters in this subgroup (Table S3).

**Table S3.** Comparison of kinematic parameters between non–weight-bearing and weight-bearing conditions in patients aged ≥35 years

|  | NWBCT  N=9  Mean ±SD | WBCT  N=9  Mean ±SD | p-value |
| --- | --- | --- | --- |
| FTR (degrees) | 9.07 ±4.52 | 3.85 ±3.20 | 0.043* |
| FTR 2 - PTTG (degrees) | 21.95 ±6.95 | 25.78 ±3.66 | 0.128 |
| Medial ATT (mm) | 4.23 ±3.39 | 6.14 ±3.49 | 0.103 |
| Lateral ATT (mm) | 3.08 ±5.34 | 1.36 ±4.90 | 0.503 |

Subgroup analysis of patients aged ≥35 years comparing femorotibial rotation (FTR), trochlear groove–patellar tendon angle (FTR 2-PTTG), and anterior tibial translation (ATT) between non–weight-bearing and weight-bearing conditions. Values are expressed as mean ± standard deviation. P-values refer to paired comparisons between conditions.

In patients aged <35 years, no statistically significant differences were found between WBCT and NWBCT conditions for any of the evaluated measurements (Table S4).

**Table S4.** Comparison of kinematic parameters between non–weight-bearing and weight-bearing conditions in patients aged <35 years

|  | NWBCT  N=7  Mean ±SD | WBCT  N=7  Mean ±SD | p-value |
| --- | --- | --- | --- |
| FTR (degrees) | 7.77 ±6.06 | 3.81 ±3.68 | 0.175 |
| FTR 2 - PTTG (degrees) | 19.76 ±6.43 | 21.05 ±5.26 | 0.606 |
| Medial ATT (mm) | 1.02 ±3.44 | 4.71 ±3.09 | 0.117 |
| Lateral ATT (mm) | 2.44 ±6.62 | 3.44 ±4.97 | 0.752 |

Subgroup analysis of patients aged <35 years comparing femorotibial rotation (FTR), trochlear groove–patellar tendon angle (FTR 2-PTTG), and anterior tibial translation (ATT) between non–weight-bearing and weight-bearing conditions. Values are expressed as mean ± standard deviation. P-values refer to paired comparisons between conditions.
